# Cumulative Effects of Nutrient Enrichment and Elevated Temperature Compromise the Early Life History Stages of the Coral *Acropora tenuis*

**DOI:** 10.1371/journal.pone.0161616

**Published:** 2016-08-30

**Authors:** Adriana Humanes, Sam H. C. Noonan, Bette L. Willis, Katharina E. Fabricius, Andrew P. Negri

**Affiliations:** 1 ARC Centre of Excellence for Coral Reef Studies, and College of Marine and Environmental Sciences, James Cook University, Townsville, Queensland, Australia; 2 Australian Institute of Marine Science, Townsville, Queensland, Australia; Instituto Español de Oceanografía, SPAIN

## Abstract

Inshore coral reefs are experiencing the combined pressures of excess nutrient availability associated with coastal activities and warming seawater temperatures. Both pressures are known to have detrimental effects on the early life history stages of hard corals, but studies of their combined effects on early demographic stages are lacking. We conducted a series of experiments to test the combined effects of nutrient enrichment (three levels) and elevated seawater temperature (up to five levels) on early life history stages of the inshore coral *Acropora tenuis*, a common species in the Indo-Pacific and Red Sea. Gamete fertilization, larval survivorship and larval settlement were all significantly reduced as temperature increased, but only fertilization was further affected by simultaneous nutrient enrichment. Combined high temperatures and nutrient enrichment affected fertilization in an additive manner, whereas embryo abnormalities increased synergistically. Higher than normal temperatures (32°C) increased coral juvenile growth rates 1.6-fold, but mortality also increased by 50%. The co-occurrence of nutrient enrichment with high temperatures reduced juvenile mortality to 36%, ameliorating temperature stress (antagonistic interaction). Overall, the types of effect (additive vs synergistic or antagonistic) and their magnitude varied among life stages. Gamete and embryo stages were more affected by temperature stress and, in some cases, also by nutrient enrichment than juveniles. The data suggest that coastal runoff events might exacerbate the impacts of warming temperatures on fertilization if these events co-occur during corals spawning. The cumulative impacts of simultaneous exposure to nutrient enrichment and elevated temperatures over all early life history stages increases the likelihood for failure of larval supply and recruitment for this coral species. Our results suggest that improving the water quality of river discharges into coastal areas might help to enhance the thermal tolerances of early life history stages in this common coral species.

## Introduction

Coral reefs around the world are facing increasing pressures from coastal human activities and climate change [1], with warming sea surface temperatures (SST) and nutrient enrichment among their most harmful stressors [2]. The simultaneous and cumulative effects of elevated SST and nutrient enrichment on demographic processes may lead to significant declines in coral cover [3]. This highlights the importance of studying their joint effects, particularly on the sensitive early life history stages that maintain and replenish coral populations, including gamete fertilization, larval supply, settlement and juvenile survivorship [4].

Since the beginning of the 20^th^ century SST has risen by a global average of ~1°C [5] and is projected to increase by a further 2 to 3°C by the end of the century under a moderate Representative Concentration Scenario of the Intergovernmental Panel on Climate Change (IPCC RCP 4.5 scenario) [6]. Such increases in SST alone would endanger many coral species, which typically live close to their upper thermal tolerance limit [7]. Additionally, coral reefs are increasingly exposed to elevated nutrients associated with terrestrial runoff from expanding agriculture and associated fertilizers and the loss of top soils [8]. Increases in the concentrations of nutrients (organic and inorganic) in a water body, which can enhance the algal production, turbidity, sedimentation of particulate matter and in severe cases can deplete oxygen concentrations is known as eutrophication [9, 10]. River runoff, resulting in the eutrophication of nearshore tropical marine habitats has been reported to cause: reductions in coral biodiversity [11], increases in macroalgae cover [12], proliferation of macro-bioeroding organisms (i.e., sponges, molluscs, polychaetes and sipunculans) that weaken the structural integrity of coral reefs [13], increases in the frequency and severity of coral diseases [14], and changes in the composition of biofilms that provide conditioned surfaces for larval settlement and metamorphosis of many sessile organisms [15–17].

Since European settlement in 1850, the development of Australia’s North Queensland catchments adjacent to the Great Barrier Reef (GBR) has led to significant changes in the quality and quantity of water discharges into the GBR lagoon [18, 19]. Expansion of agricultural and grazing activities, the clearing of vegetation leading to widespread soil erosion, and the application of fertilizers has increased river discharges of dissolved and particulate organic and inorganic nutrients and trace elements in the region [8, 18, 20, 21]. Inorganic nutrients from anthropogenic sources generally only persist in the GBR lagoon for periods of days to weeks [20], as they are rapidly taken up by microbial and planktonic communities. They are then transformed into organic matter and undergo complex cycling between particulate and dissolved forms, organic and inorganic forms, and undergo repeated deposition-resuspension cycling [22, 23]. Recent studies estimate that nutrient loads of rivers discharging into the GBR lagoon have increased by a factor of 5.7 for nitrogen and 8.9 for phosphorus since European settlement [18], leading to significant organic enrichment in inshore waters [24]. For the foreseeable future, coastal marine ecosystems are likely to face further increases in eutrophication (inorganic and organic enrichment) as a consequence of nutrient inputs from river runoff [25], as well as increases in SST due to climate change [6].

Reproduction and early life history stages of marine organisms can be particularly vulnerable to environmental stress [26]. Most scleractinian corals are broadcast spawners, simultaneously releasing buoyant eggs and sperm into the water column for external fertilization [27, 28]. Spawning and larval development of the majority of coral species on the GBR takes place in early summer (October to December)[29] can coincide with nutrient discharges typically driven by major river flood events during the summer monsoonal wet season (October to April)[30]. Co-occurrence of heat stress and floods with broadcast spawning would place the sensitive early life history stages of hard corals (gametes, embryos, larvae and recruits) at risk. Despite the perception that early life history stages of corals are more sensitive to environmental change and pollution than adult stages [12], few studies have empirically addressed their susceptibility to the co-occurrence of multiple pressures [31–35].

Several studies on tropical coral species of the Caribbean and the Indo-Pacific have demonstrated detrimental impacts of nutrient enrichment [12, 36, 37] or elevated seawater temperatures [38–42] on coral reproduction, growth, health and survivorship. Moreover, eutrophication renders adult corals more susceptible to thermal bleaching, as nutrient enrichment enhances the abundance of algal symbionts [43], increasing the ratio of symbiont to host cells, which can increase the vulnerability of this symbiotic partnership to disruption associated with high sea temperatures [36, 44, 45]. While evidence is mounting that interactions between elevated SST and nutrient enrichment might have important deleterious effects at the population level [46–48], no studies have investigated the combined effects of these stressors on the early life history stages and processes of corals (from gamete fertilization to coral juveniles).

An improved understanding of how present and future combinations of stressors are likely to affect early life history stages of hard corals is needed to adequately assess and develop management policies for coral reef ecosystems [49]. Here we describe a series of experiments that tested the effects of elevated temperature and nutrient enrichment (mimicking eutrophication) on the fertilization success of coral gametes, development and settlement of coral larvae, and the growth, photophysiology and survivorship of 4-month-old coral juveniles. The study was conducted with the common inshore coral species *Acropora tenuis*, and aimed to: 1) understand the combined effects of elevated temperature and nutrient enrichment when they co-occur; 2) identify the most sensitive early life history stages to elevated temperatures and nutrient enrichment, and 3) provide a minimum estimate of their combined effects on population replenishment.

## Materials and Methods

### Obtaining coral gametes and juveniles

Gravid colonies (> 20 cm diameter) of the broadcast spawning coral *Acropora tenuis* (Dana, 1846), an abundant species on shallow inshore coral reefs of the GBR, were collected from Magnetic Island (19° 06’S, 146° 51’E) at ~6 m depth on the 5^th^ of November 2014 under the permit G12/35236.1 issued by the Great Barrier Reef Marine Park Authority. Colonies were transferred to outdoor flow-through temperature-controlled aquaria at the National Sea Simulator at the Australian Institute of Marine Science (AIMS), where seawater temperatures were set to ambient reef temperatures on the day of collection (27°C). Following spawning, 5 days after full moon (at ~19:30) egg-sperm bundles were gently scooped from the surface of the water, and eggs were separated from sperm using a 100 μm mesh filter and gently washed five times in 0.2 μm filtered sea water (FSW), as described in Negri and Heyward [50]. Concentrated sperm water was diluted to achieve a working stock mixture of ~1 x 10^7^ sperm ml^-1^ to optimise fertilization success [51]. A subsample of gametes was used for the fertilization experiment (Experiment 1), and the remaining gametes were mixed and fertilized [50]. Bulk larval cultures were reared for the larval settlement and juvenile experiments (2 and 3) in 500 l flow-through tanks using 1 μm-filtered seawater at 27°C.

### Experimental setups

**Fig 1:**
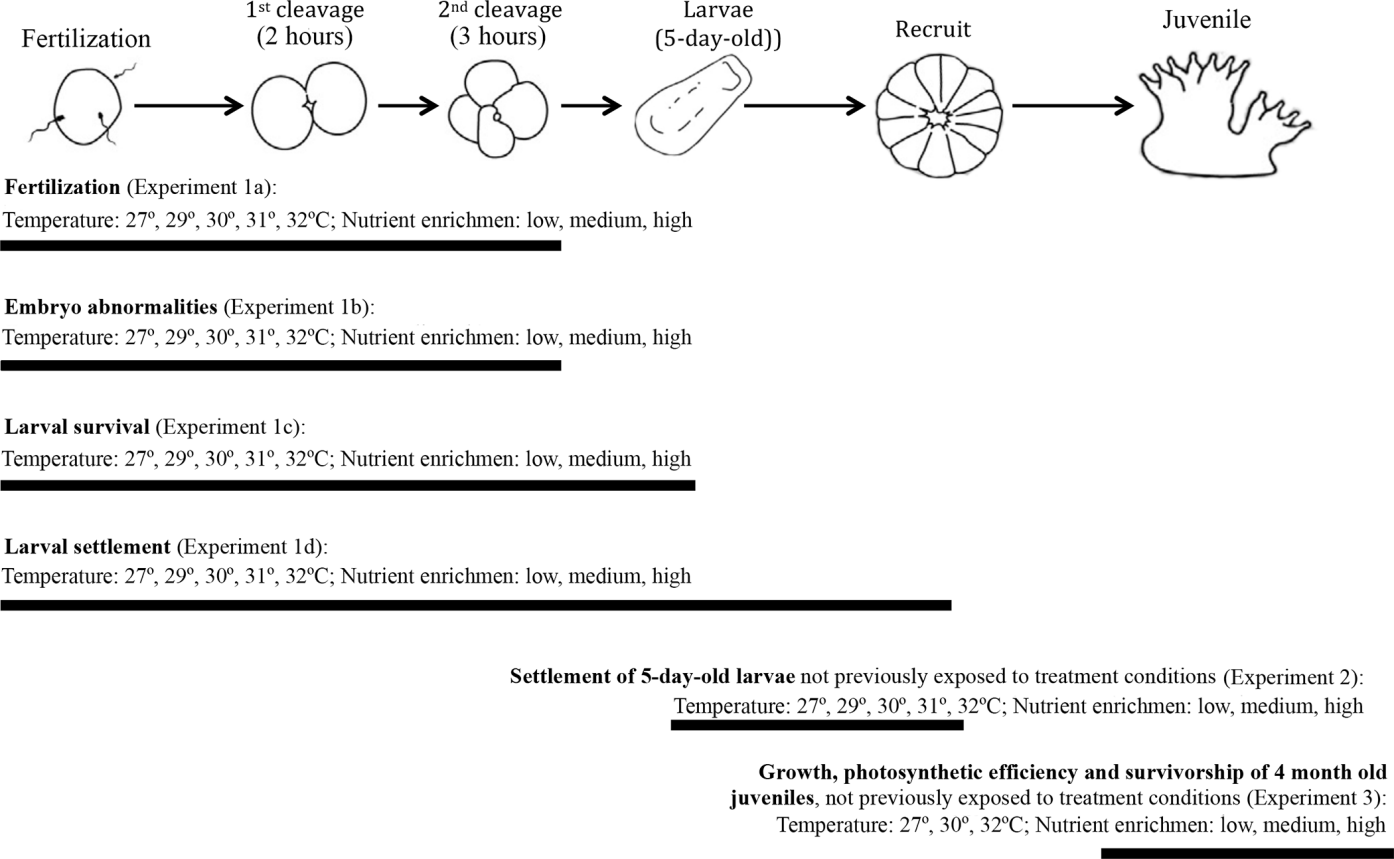
Experiments performed with early life history stages of *Acropora tenuis* exposed to different treatments of temperature and nutrient enrichment. Black bars indicate the stages involved in each experiment.

**Fig 2:**
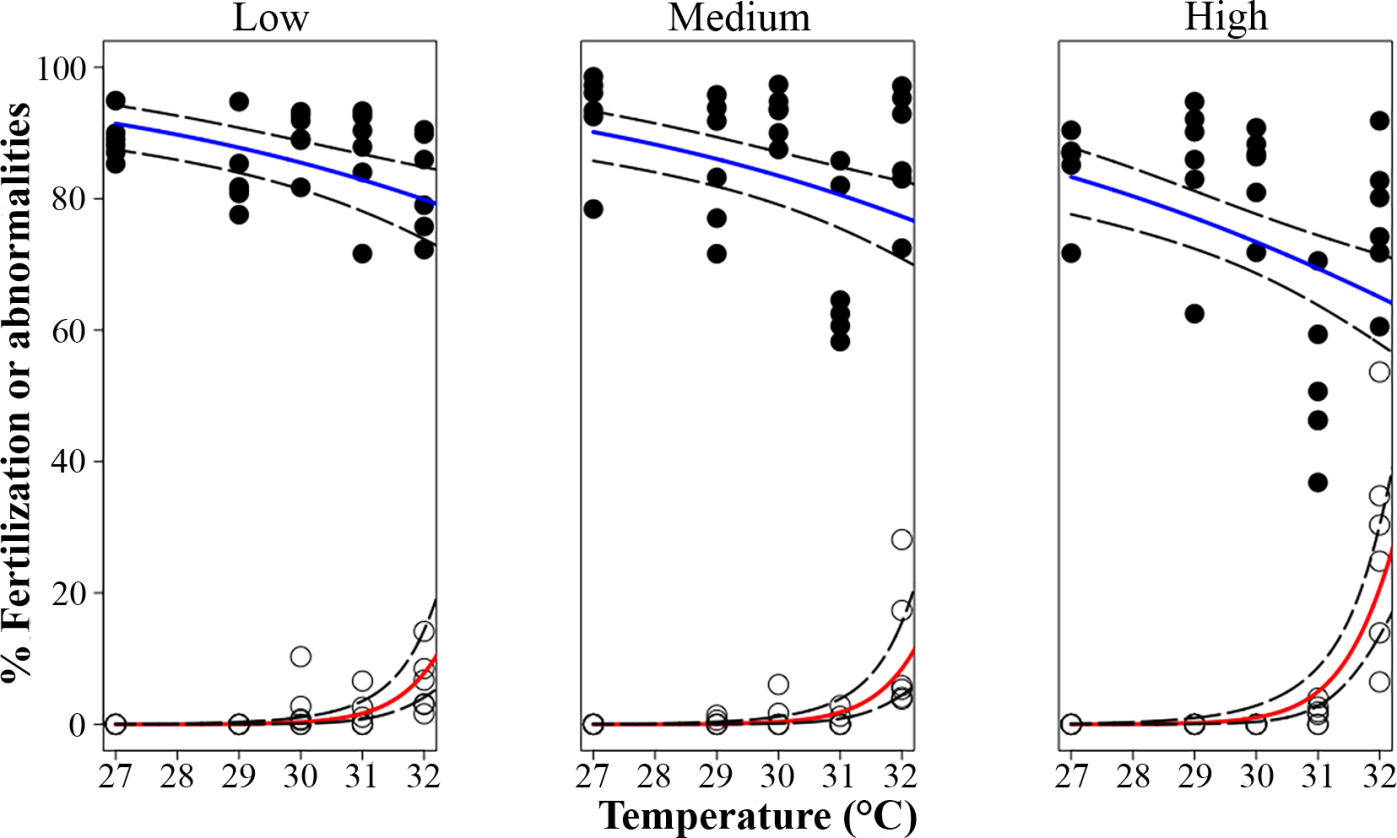
**Effects of temperature and nutrient enrichment on the percentage of fertilized eggs (blue line, black circles; Experiment 1a) and abnormal embryos (red line, open circles; Experiment 1b) of *Acropora tenuis*.** Control treatment: ‘low’ nutrient enrichment and at temperature = 27°C. Solid lines indicate fitted GLM trend lines, while dashed lines are 95% confidence intervals.

**Fig 3:**
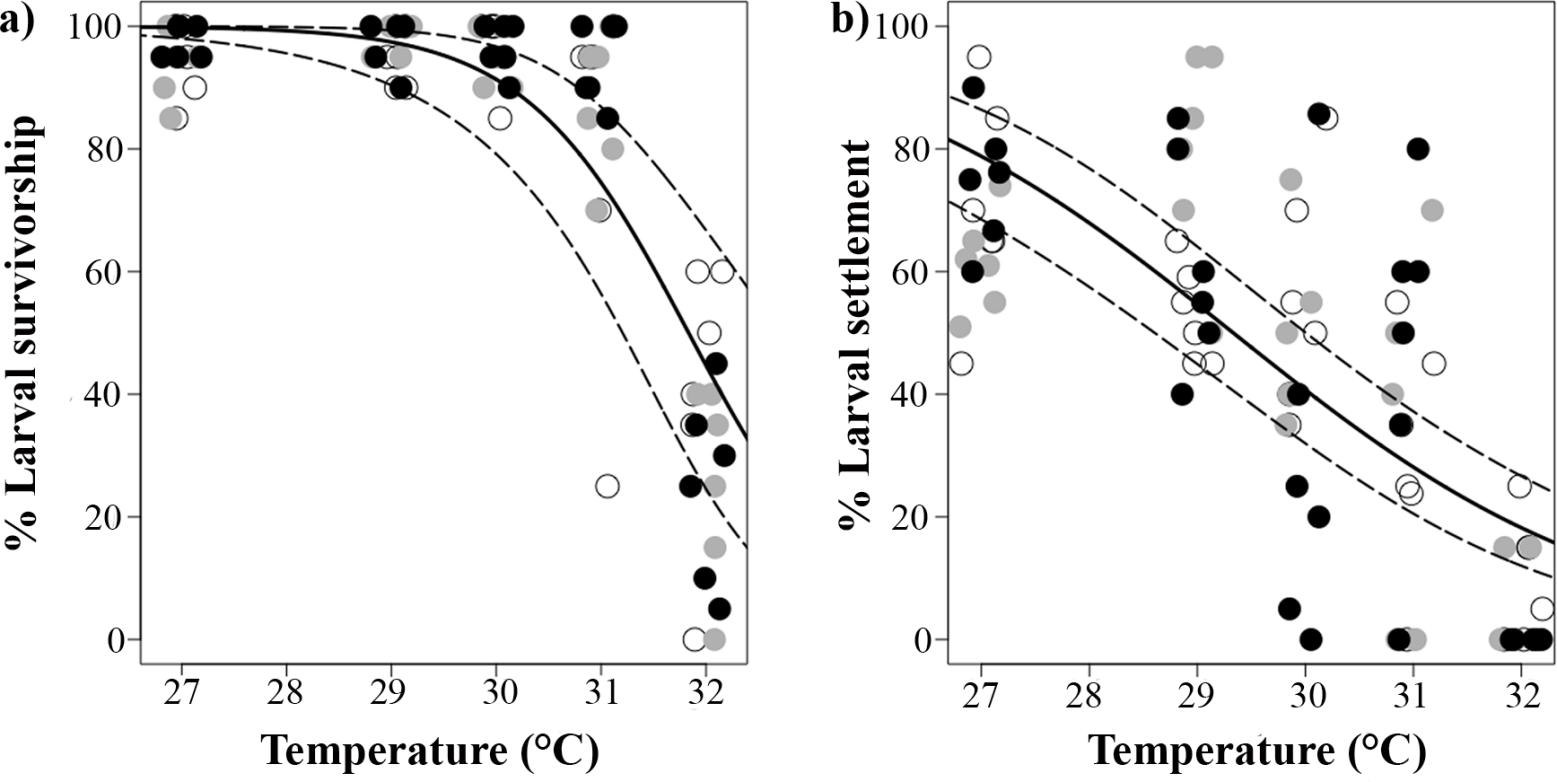
**a) Percentage larval survivorship 5 days after fertilization for *Acropora tenuis* reared under different temperatures and nutrient enrichment [low (open circles), medium (grey circles), high (black circles); Experiment 1c]. b) Settlement rates for larvae of *A*. *tenuis* that had being fertilized, reared and settled under different temperatures and nutrient enrichment (Experiment 1d).** Control treatment: ‘low’ nutrient enrichment and at temperature = 27°C. Solid lines indicate fitted GLM trends, while dashed lines are 95% confidence intervals. Individual points are jittered horizontally for clarity.

Three experiments were conducted to investigate the combined effects of nutrient enrichment (organic and inorganic nutrient enrichment) and elevated seawater temperatures on early life history stages and processes (from gamete fertilization to 4-month-old juveniles) of *A*. *tenuis*. Experiments were designed to mimic the impact of nutrient enrichment as a consequence of river plumes and terrestrial runoff events, which wash nutrients and trace elements onto inshore reefs, where they are taken up by plankton communities and converted into organic matter. Experimental concentrations of nutrients were chosen to lie within the range of those measured on inshore GBR reefs [18, 52, 53]. Temperature treatments corresponded to increases of +2 to +5°C above ambient temperatures recorded during coral spawning periods on reefs of the GBR [54]. Experiments were conducted to test the effects of nutrient enrichment (three levels) together with temperature (up to five levels) on: 1) gamete fertilization, embryo development and larval settlement (Experiment 1, Fig 1), 2) settlement of 5-day-old larvae when no preceding stages were exposed to treatment conditions (Experiment 2, Fig 1) and 3) the photophysiology, growth, and survivorship of 4-month-old coral juveniles when no preceding stages were exposed to treatment conditions (Experiment 3, Fig 1). The small size of recently settled coral recruits (~1 mm) makes it difficult to measure physiological variables, and they were therefore allowed to grow for four months before commencing this experiment.

#### Preparation of nutrient enrichment and temperature treatments

For Experiments 1 and 2, nutrient enrichment treatments were prepared by adding inorganic and organic nutrients derived from inshore organic matter and plankton to FSW. Inshore nutrients and plankton were collected with a plankton net (mesh size 100 μm) over the reefs of Orpheus Island (18° 36’S, 146° 29’E). On the inshore of the GBR, suspended particulate matter mostly consists of decayed detritus resuspended from the seafloor, and zooplankton [55]. The bulk material caught with the net was sieved to remove large fragments (> 26 μm), homogenized with a blender and frozen until use. Two replicate glass Schott bottles (each 2 l) were used to incubate the nutrient enriched seawater for each treatment for 48 h at 200-μmol photons m^-2^ s^-1^ light intensity (12 h:12 h diurnal cycle) at the target temperatures [27, 29, 30, 31 and 32°C, S1 Table]. Incubations were conducted to allow the microbial communities to take up the bioavailable fraction of the inorganic nutrients and transform it into organic nutrients [56], a process that is accelerated as temperatures increase [57]. Previous studies have successfully applied this method for studying the effects of nutrient enrichment on corals [24, 58], with incubations of 48 h being required for the microbial community to develop [59–61]. Nutrient enrichment treatments were performed using decaying natural plankton in order to maintain a realistic stoichiometric composition of nutrients and trace elements. Based on the total organic carbon (OC) present in the collected nutrient-plankton mixture, three nutrient enrichment treatments were prepared by adding the required volume of the mixture to FSW, at a nominal concentration of +0, +0.3, or +0.6 mg OC l^-1^ FSW. Such organic carbon enrichment levels are environmentally relevant for river-influenced inshore reefs in the GBR ([53], Table 1).

**Table 1:** Water quality parameters for the different temperatures and nutrient enrichment (low in white, medium in light grey and high in dark grey) at the start of each experiment. Values shown are means and standard deviations. Number of replicates: 2 per water quality factor and treatment for Experiments 1 and 2, 18 per treatment for Experiment 3 at 27°C, and 6 per treatment for Experiment 3 at 30 and 32°C temperature. Ranges from seawater values from the inshore of the Great Barrier Reef are added for comparison [53].

Experiment	Temperature (°C)	Nutrients	DOC (μM)	TOC (μM)	NH_4_ (μM)	NO_2_+NO_3_ (μM)	NO_2_ (μM)	TDN (μM)	TN (μM)	PO_4_ (μM)	TDP (μM)	O_2_ (mg l^-1^)
Schaffelke et al 2012 [53]			42.8–195.7	3.9–70.5	0–0.8			2.3–11.5	0.5–2.8	0.02–0.6	0–1.01	
1a, b	27	Low	84.1±0.1	8.5±3.6	0.5±0.1	0.5±0.1	0.2±0.1	8.8±0.5	0.9±0.1	0.1±0.1	0.2±0.1	8.0±0.2
		Medium	92.5±4.0	8.7±1.8	4.4±0.1	1.3±0.1	0.8±0.1	15.7±1.6	1.2±0.1	0.4±0.1	0.5±0.1	7.8±0.1
		High	99.2±4.8	8.7±3.4	8.9±0.1	1.4±0.1	0.8±0.1	23.0±0.1	1.5±0.1	0.7±0.1	0.7±0.1	7.7±0.1
	29	Low	86.3±2.6	5.0±3.4	0.8±0.4	0.7±0.1	0.2±0.1	9.3±0.2	0.8±0.3	0.1±0.1	0.2±0.1	7.9±0.1
		Medium	91.2±0.2	7.7±2.7	4.4±0.1	1.3±0.1	0.7±0.1	15.9±0.8	1.5±0.3	0.4±0.1	0.5±0.1	7.6±0.1
		High	92.9±5.3	9.6±6.1	9.2±0.1	1.4±0.1	0.8±0.1	22.4±1.6	1.8±0.4	0.6±0.1	0.7±0.1	7.8±0.3
	30	Low	91.5±0.1	2.3±1.2	0.7±0.1	0.8±0.1	0.2±0.1	10.2±0.6	0.8±0.1	0.1±0.1	0.3±0.1	7.8±0.1
		Medium	92.5±1.6	5.3±1.2	4.6±0.1	1.2±0.1	0.5±0.1	15.4±0.7	1.5±0.3	0.4±0.1	0.6±0.1	7.7±0.1
		High	89.1±6.3	8.0±1.7	9.4±0.1	1±0.1	0.4±0.1	22.0±1.3	1.6±0.4	0.7±0.1	0.8±0.1	7.6±0.1
	31	Low	84.8±3.5	3.9±2.4	0.7±0.1	0.6±0.1	0.1±0.1	9.8±1.0	0.9±0.5	0.1±0.1	0.3±0.1	7.6±0.1
		Medium	90.0±3.2	5.2±1.3	4.7±0.2	1±0.1	0.4±0.1	15.7±0.6	1.3±0.3	0.4±0.1	0.5±0.1	7.7±0.1
		High	93.8±6.8	8.3±2.8	9.6±0.3	1.1±0.1	0.4±0.1	21.9±0.5	1.9±0.5	0.7±0.1	0.9±0.1	7.6±0.1
	32	Low	90.7±2.5	4.3±3.1	0.7±0.1	0.8±0.1	0.1±0.1	10±0	1.1±1.1	0.2±0.1	0.3±0.1	7.7±0.1
		Medium	90.8±2.9	5.0±0.2	4.6±0.3	1.1±0.1	0.4±0.1	14.9±0.3	1.4±0.1	0.4±0.1	0.5±0.1	7.6±0.1
		High	95.7±1.8	8.2±2.7	9.7±0.2	1.0±0.1	0.3±0.1	22.2±1.0	1.7±0.4	0.5±0.1	0.9±0.1	7.5±0.1
1c, d, 2	27	Low	76.8±2.8	8.3±2.0	0.8±0.1	0.6±0.1	0.3±0.1	10.7±0.9	1.7±0.1	0.1±0.1	0.3±0.1	8.2±0.1
		Medium	85.9±2.4	19.7±3.5	4.5±0.2	0.6±0.1	0.3±0.1	16±0.5	5.0±0.7	0.3±0.1	0.5±0.1	7.7±0.1
		High	87.5±7.6	23.2±1.3	6.1±0.3	0.6±0.1	0.3±0.1	18.2±1.3	6.2±0.4	0.4±0.1	0.6±0.1	7.6±0.1
	29	Low	102.9±25.4	6.8±0.6	0.8±0.1	0.6±0.1	0.3±0.1	11.7±0.7	1.6±0.3	0.2±0.1	0.4±0.1	7.9±0.1
		Medium	111.9±32.7	25.7±1.7	5.0±0.1	0.6±0.1	0.3±0.1	17.2±0.3	4.3±0.1	0.3±0.1	0.5±0.2	7.7±0.1
		High	120.2±39.8	34.8±6.0	7.5±0.1	0.7±0.1	0.4±0.1	20.2±0.6	6.3±0.5	0.5±0.1	0.6±0.1	7.6±0.1
	30	Low	82.9±0.2	11.3±0.9	0.9±0.1	0.6±0.1	0.3±0.1	11.4±0	1.7±0.1	0.2±0.1	0.4±0.1	7.7±0.1
		Medium	83.1±4.1	21.3±6.4	4.8±0.3	0.6±0.1	0.2±0.1	16.4±1.9	5.4±1.5	0.4±0.1	0.5±0.1	7.6±0.1
		High	87.6±3.8	21.6±3.5	7.9±0.2	0.6±0.1	0.3±0.1	20.0±0.5	5.3±1.2	0.5±0.1	0.7±0.1	7.5±0.1
	31	Low	82.3±2.9	7.2±1.6	0.7±0.0	0.6±0.1	0.1±0.1	9.8±1.0	0.9±0.1	0.1±0.1	0.3±0.1	7.7±0.1
		Medium	86.4±5.9	17.9±1.7	5.1±0.1	0.5±0.1	0.2±0.1	16.8±0.3	3.8±0.3	0.4±0.1	0.4±0.1	7.7±0.1
		High	87.4±5.4	25.6±1.2	8.1±0.8	0.6±0.1	0.3±0.1	21.1±0.2	5.7±0	0.6±0.1	0.8±0.1	7.6±0.1
	32	Low	101.9±16.2	7.7±NA	0.9±0.1	0.6±0.1	0.3±0.1	11.0±0.3	1±NA	0.2±0.1	0.2±0.1	7.6±0.1
		Medium	102.7±23.3	20.3±2.2	5.0±0.0	0.6±0.1	0.2±0.1	16.6±0.1	3.9±0.1	0.4±0.1	0.5±0.1	7.6±0.1
		High	154.9±95.6	27.2±NA	8.7±0.8	0.6±0.1	0.3±0.1	22.5±2.2	6.3±NA	0.6±0.1	0.7±0.1	7.5±0.1
3	27	Low	152.1±25.5	45.2±28.5	4.3±9.3	0.2±0.1	0.1±0.1	32.1±39.2	7.1±4.3	0.1±0.1	0.2±0.1	8.0±0.2
		Medium	167.2±68.3	64.7±34.0	7.3±10.4	0.2±0.1	0.1±0.1	35.9±24.7	11.6±7.3	0.3±0.4	0.5±0.6	7.8±0.1
		High	173.9±36.6	74.6±51.8	15±23.3	0.3±0.4	0.1±0.1	44.8±41.8	14.5±12	0.7±0.9	0.9±1.1	7.6±0.2
	30	Low	160.3±50.1	35.7±30.5	0.6±0.4	0.1±0.1	0.1±0.1	14.5±2.8	5.6±3.5	0.1±0.1	0.2±0.1	7.7±0.1
		Medium	161.5±23.2	42.7±32.4	0.9±0.8	0.1±0.1	0.1±0.1	18.4±8.1	7.0±5.4	0.1±0.1	0.2±0.1	7.7±0.1
		High	187.3±31.8	50.8±23.5	1.2±1.6	0.1±0.1	0.1±0.1	39.5±38.8	7.6±3.3	0.1±0.1	0.2±0.1	7.6±0.1
	32	Low	159.3±37.7	44.5±24.0	1.7±2.1	0.1±0.1	0.1±0.1	22.5±8.8	8.0±4.0	0.1±0.1	0.2±0.1	7.6±0.1
		Medium	171±31.7	53±35.2	5.3±9.1	0.1±0.1	0.1±0.1	56.1±37.3	9.4±7.8	0.1±0.1	0.2±0.1	7.7±0.3
		High	178.3±39.4	63±37.4	6.4±7.8	0.2±0.1	0.1±0.1	80±76.4	10.3±8	0.1±0.1	0.3±0.1	7.6±0.1

In Experiment 3, nutrient enriched treatments were prepared and incubated in 4 l polyethylene tanks with gentle aeration. Tanks were placed in temperature-controlled water baths (six tanks per water bath, at temperatures: 27, 30 and 32°C, S1 Table) and illuminated as in Experiment 2. Coral juveniles were kept in 18 gently aerated experimental tanks (4 l) in water baths (at temperatures: 27, 30 and 32°C), but under 60 μmol photons m^-2^ s^-1^ light. Seawater in the experimental tanks was replaced every two days with seawater that had been enriched with nutrients and incubated over the previous 48 h.

To characterize water quality of the different nutrient enrichment treatments, concentrations of total organic carbon, dissolved organic carbon, particulate organic carbon, total dissolved phosphorus, dissolved organic nitrogen, total particulate nitrogen, ammonium, phosphate_,_ nitrate, and nitrite, were measured at the end of the incubation in duplicate subsamples from all Schott Bottle replicates in Experiments 1 and 2, and on a weekly basis after incubation in all replicates in Experiment 3. In this way, water quality parameters of the different treatments were always measured after the incubations, at the start of each experiment. Water quality samples were taken following standard protocols as described in detail in Schaffelke et al. [53] and analysed by the Analytical Services laboratory at AIMS.

The addition of nutrients to FSW increased the concentrations of all water quality variables measured in each of the three Experiments (Table 1). Nutrient concentrations varied after the incubation between experiments, therefore nutrient enrichment treatments were designated as ‘low’, ‘medium’ and ‘high’ nutrient enrichment, corresponding to the addition of +0, +0.3, and +0.6 mg OC l^-1^ FSW. In all experiments the treatment with ‘low’ nutrient enrichment and at temperature = 27°C was considered as the control.

### Elevated temperature and nutrient enrichment effects on gamete fertilization, embryo development and larval settlement (Experiment 1)

Fertilization experiments (Experiment 1a, Fig 1, Table 2) were conducted in six-well polystyrene tissue culture plates (NuncTM, Denmark), with each treatment having six replicate wells. A total of 15 treatments were established, with three levels of nutrient enrichment (+0, +0.3, +0.6 mg OC l^-1^ FSW) and five levels of temperature (27, 29, 30, 31 and 32°C). Plates were maintained in temperature incubators (S1 Table) 60 min before the start of each experiment and throughout the duration of the experiment. Duplicate plates (n = 12 wells) containing 6 ml of the nutrient enriched seawater, combined with either ~170 eggs or 1 ml stock sperm mixture, were prepared for each treatment in order to pre-expose gametes separately for 30 min before combining them to initiate fertilization. The final sperm concentration was 5x10^4^ sperm ml^-1^, being slightly suboptimal for maximum fertilization [51], thereby increasing the sensitivity of the assay [62, 63]. When the third cleavage was observed (after ~2.5 h), 2 ml of buffered zinc formalin fixative (Z-fix preservative, Anatech Limited) were added to terminate embryo development and preserve embryo integrity.

**Table 2:** Experimental conditions used in each experiment performed at different temperatures and nutrient enrichment.

Experiment	Nutrient enrichment	Temperature (°C)	Treatment volume (ml)	Replicates	Exposure time	Stage exposed	Number of individuals per replicate	Variable measured
1a	Low, medium, high	27, 29, 30, 31, 32	12	6	2.5 hours	Gametes	~170 eggs	% Fertilization
1b	Low, medium, high	27, 29, 30, 31, 32	12	6	2.5 hours	Gametes		% Abnormalities
1c	Low, medium, high	27, 29, 30, 31, 32	40	6	5 days	Larvae	20 larvae	% Larvae survivorship
1d	Low, medium, high	27, 29, 30, 31, 32	10	6	6 days	Larvae	10 larvae	% Larvae settlement
2	Low, medium, high	27, 29, 30, 31, 32	10	12	1 day	Larvae	10 larvae	% Larvae settlement
3	Low, medium, high	27, 30, 32	4000	2	59 days	Juveniles	19 juveniles	Growth, production of new polyps, final weight F_v_/F_m_, survivorship

Early embryo development (Experiment 1b, Fig 1, Table 2) was assessed using a stereomicroscope, and fertilization success (proportion of eggs fertilized) and embryo quality (proportion of normal versus abnormal embryos developing from the fertilized gametes) were recorded. Coral embryos were considered normal if they underwent radial holoblastic cleavage, with regular cleavage patterns until the eight-cell stage, which generally occurred within 3–8 h [64]; abnormal embryos deviated from this division pattern, resulting in asymmetrical development and/or fragmentation.

Embryos (2.5 h old) that were developing normally in the same 15 treatments were selected to test larval survivorship (Experiment 1c, Fig 1, Table 2). For each temperature-nutrient combination, 20 embryos were incubated at 60 μmol photons m^-2^ s^-1^ in 50 ml polypropylene jars containing 40 ml of treatment seawater (as above, n = 6 jars per treatment). Water changes with new enriched seawater were performed 48, 72, and 96 h after fertilization. Larval survivorship was assessed on day 5, when larvae show active swimming movements, display settlement behaviour by testing the substratum for settlement cues, and become competent to settle [27]. Larvae were counted and transferred to six-well plates with 10 ml of treatment seawater (n = 6 replicate wells per treatment; 1–10 larvae per well depending on larval survivorship). To induce larval settlement, 2 mm^2^ chips of live *Porolithon onkodes*, *a* crustose coralline algae (CCA), were added to each well [65]. Chips were prepared using bone cutters 1 h before adding larvae to the wells, and were obtained from a single 10 cm^2^ fragment of CCA that had been maintained in a 400 l flow-through tank at 27°C with low light intensity (60-μmol photons m^-2^ s^-1^ over a 12:12 diurnal cycle). Special care was taken during the maintenance of CCA fragments (i.e. algae removed with a toothbrush when necessary), and their ability to induce settlement was tested 18 h before using them in the experiment by offering chips of the same fragment to larvae fertilized and reared under control conditions (n = 6 replicate wells, 10 larvae per well, settlement success = 98%). After 24 h, the number of metamorphosed larvae in each well was recorded (Experiment 1d, Fig 1).

### Elevated temperature and nutrient enrichment effects on the settlement of 5-day-old larvae (no preceding stages exposed to treatment conditions; Experiment 2)

The settlement of 5-day-old larvae that had not previously been exposed to elevated temperatures or nutrients (i.e. raised under control conditions) were used to assess the effects of nutrient enrichment (+0, +0.3, +0.6 mg OC l^-1^ FSW) and temperature (27, 29, 30, 31 and 32°C) on the process of larval settlement (Experiment 2, Fig 1, Table 2). Six-well plates were maintained in temperature incubators for 60 min before the start of each experiment to reach treatment temperatures (S1 Table) and during the experiment. Ten larvae were added to each well of two six-well polystyrene tissue culture plates (NuncTM, Denmark), for each of the three nutrient-enriched seawater treatments (n = 12 wells/treatment; seawater enriched as per Experiment 1). CCA chips (as above) were added to each well and settlement success was assessed after 24 h.

### Elevated temperature and nutrient enrichment effects on the physiology and survivorship of 4-month-old coral juveniles (Experiment 3)

Four-month-old juveniles of *A*. *tenuis* (1–11 polyps) individually settled on manufactured aragonite substrata (~2 cm in diameter) commonly used by aquarists (Oceans Wonders LLC), were exposed to three levels of both nutrient enrichment (+0, +0.3, +0.6 mg OC l^-1^ FSW) and temperatures (27, 30 and 32°C, Fig 1, Table 2). Prior to the experiment and during all their preceding life stages, juveniles were kept at 27°C and ambient nutrient conditions. Two replicate tanks (each 4 l) were set up for each of the nine treatments, and 19 juveniles were added to each of the 18 tanks. The juveniles were exposed to the three nutrient enrichment treatments at 27°C for 20 days before starting the temperature stress. For the heat stress, external sensor-controlled heat exchange units were used to warm the water in four water baths (two used for the incubation of the nutrient enrichment FSW, and two for the experimental tanks, S1 Table). Temperature was ramped up from 27 to 30 or 32°C over a 2-day period. Water temperature was measured daily in all tanks, and temperature loggers were used in each experimental water bath housing the tanks. Once established, juveniles were kept under treatment conditions for a further 37 days, by which time half of the juveniles in the highest temperature treatment (32°C) had died and measurable differences had been detected among treatments for most of the variables.

#### Photochemical efficiency of the symbionts

Maximum quantum yield (F_v_/F_m_) of photosystem II (PSII), a measure of the proportion of available light that can be photochemically quenched, was measured for all surviving juveniles on day 54 of the experiment. A reduction of F_v_/F_m_ is indicative of photooxidative stress and damage to PSII [66]. Measurements of F_v_/F_m_ were made using a Maxi Imaging Amplitude Modulation Fluorometer (I-PAM, Walz GmbH, Germany), which measures the fluorescence of a selected area of interest in an image (i.e., the juvenile). Measurements were performed by placing all surviving juveniles from each treatment tank into a 0.5 l container under the treatment conditions. Juveniles were dark-adapted for one hour prior to each saturation light pulse (gain = 1, intensity = 7, saturation pulse = 5) and F_v_/F_m_ calculated using the formula F_v_/F_m_ = (F_m_−F_0_)/F_m_ with F_v_ = variable fluorescence, F_m_ = maximum fluorescence, and F_0_ = minimum fluorescence [67].

#### Survivorship, growth and weight

Survivorship of juveniles was assessed every two days by placing each recruit in a 60 ml chamber filled with the treatment water and observing it using a stereomicroscope. Death was defined as the time point when live tissue was no longer present. Survivorship was expressed as the proportion of colonies within each tank that survived to day 59 (20 days of nutrient exposure + 2 days of temperature ramping + 37 days nutrient and temperature exposure) in relation to the initial number of juveniles at the beginning of the experiment (19 juveniles per tank). The number of polyps per juvenile was counted on days 4 and 59. Images of each juvenile were taken on days 39 and 59, using a Leica MC170 stereomicroscope. The area of live tissue was measured with the program ToupView 3.7 and was used as an estimate of the colony size. At this age, juvenile morphology was typically 2-dimensional, enabling a good estimation of their planar surface areas (size). Growth (μm^2^ day^-1^) was estimated as the change in area of each juvenile colony over 20 days (between day 39 and 59 of the experiment). On day 59, the juveniles were carefully detached from the substrate using a needle, and placed in 20 ml scintillation vials with chlorine 6% for 3 days. Skeletons were washed twice with Milli-Q water and dried at 60°C for 48 hours in an oven before final weight measurements were taken with a microbalance.

### Data analysis

Generalized linear models (GLM) were used to assess changes in fertilization success, embryo development, larval survivorship and settlement as a function of temperature (fixed numerical factor) and nutrient enrichment (fixed categorical factor). Quasi-binomial errors and the log link function were used when models had overdispersion. Linear mixed effects models were used to model changes in growth rates, the production of new polyps, the ratio of final weight to final size of coral juveniles, and photochemical efficiency of symbiotic *Symbiodinium* (F_v_/F_m_) with both temperature and nutrient enrichment as fixed factors (categorical) and tank as random error term. Survivorship curves of coral juveniles were estimated using the Kaplan-Meier method [68], a non-parametric statistic that estimates survivorship conditional probabilities at each time point. Survivorship curves were compared using an accelerated failure time model with a Weibull distribution. The analyses were conducted with the lme4 and the survival packages in R (R Development Core Team, 2016). A multiplicative model was used to determine the type of effects (additive, multiplicative synergistic or multiplicative antagonistic) in all analysis except for the response variable F_v_/F_m_ for which an additive model was used since data were normally distributed and no transformation was required [69].

To determine the total effect size (SEF_total_) of the simultaneous exposure to the two factors, the effect sizes (SEF) of all individual factors and their interactions were expressed as the proportion of change in the response variable evaluated (i.e., fertilization, larval survivorship and settlement, and juvenile survivorship) compared to control conditions (27°C, +0 mg l^-1^ OC). In order to estimate the total effect of exposing several early stages to combined temperature and nutrient enrichment, the following equation was used:

$$SEF_{total}=100*\left[\left(1-SEF_{i}\right)*\left(1-SEF_{i+1}\right)*\left(1-SEF_{n}\right)\right]$$ (1)

where SEF_total_ denotes the size (percentage) of the total effect on the final process considered (*i*.*e*. recruit success), SEF_*i*_ denotes the size of the effect of the stressors (proportion) on a particular process (*i*.*e*. fertilization), and n denotes the number of stages. SEF_total_ values can vary between 0 and +∞. SEF_total_ = 0 indicates the maximum treatment effect (i.e. 0 recruit success), SEF_total_ = 100 indicate no treatment effect, and SEF_total_ < 100 and SEF_total_ > 100 indicate a negative and positive effect of the treatment, respectively (*i*.*e*. SEF_total_ = 80 represents a decrease of recruitment by 20%, and SEF_total_ = 150 indicates an increase of 50% in recruitment). The SEF_total_ was estimated for early life history stages that had been exposed to treatment conditions for gamete fertilization, embryo development, larval survivorship and settlement, and the survivorship of 4-month-old juveniles. This represents a minimum estimation of the total effect, because the stages between recently settled larvae and 4-month-old juveniles were not exposed to the temperature and nutrient treatments and are therefore considered to be constant.

## Results

### Elevated temperature and nutrient enrichment effects on gamete fertilization (Experiment 1a)

Fertilization success was high (83 ± 6%, mean ± sd) across all temperatures and nutrient enrichment treatment combinations up to 30°C (Fig 2). There were significant detrimental main effects from both temperature elevation and nutrient enrichment on fertilization success (p <0.001, Table 2; Fig 2), while the interactions were non-significant, indicating additivity of effects on the log scale (p_Temperature*Nuttrients_ = 0.389 Fig 2, Table 2). The reduction in fertilization success compared to the control treatment (nutrient enrichment = ‘low’, temperature = 27°C) was 5 ± 6% at nutrient enrichment = ‘high’ (temperature = 27°C), and 8 ± 7% at temperature = 32°C (nutrient enrichment = ‘low’), while temperature = 32°C and nutrient enrichment = ‘high’ in combination resulted in a 14 ± 10% decline in fertilization.

### Elevated temperature and nutrient enrichment effects on embryo development and larval settlement (Experiment 1b)

Elevated temperature and nutrient enrichment together increased the proportions of abnormal embryos (Fig 2) in an interactive fashion (p_Temperature*Nutrients_ < 0.001, Table 3). Between 27 and 29°C, 100% of embryos underwent normal development characterized by radial holoblastic cleavage, resulting in equally-sized blastomeres, regardless of nutrient enrichment. Abnormalities in the form of asymmetrical and irregular cleavage increased in the ‘high’ nutrient enrichment (6 ± 12%), while the individual effect of the highest temperature (32°C) was a pronounced increase (15 ± 14%). When the highest levels of both factors co-occurred, the proportion of abnormal embryos increased (27 ± 16%) to values higher than expected for the addition of the individual effects of temperature and nutrient enrichment (Fig 2), indicating a multiplicative synergistic interaction of both stressors according to the GLM model with the log-link function.

**Table 3:** Results of treatments effects on *Acropora tenuis* early life history stages based on generalized linear models (GLM) with log-link function temperature (Temp) and nutrient enrichment (Nut) as fixed factors and tank as random error term. Significance at p<0.05 is shown in **bold**. Refer to S2 Table for detailed information for the analyses.

Experiment	Dependent variable	Treatment effect	p values		
			Temp	Nut	Temp x Nut
1a	Fertilization	Temp and Nut decreased fertilization	**<0.001**	**<0.001**	0.389
1b	Abnormalities	Temp and Nut increased abnormalities	**<0.001**	**<0.001**	**<0.001**
1c	Larval survivorship	Temp decreased larval survivorship	**<0.001**	0.880	0.181
1d	Settlement	Temp decreased settlement	**<0.001**	0.951	0.895
2	Settlement	Temp increased settlement	**<0.001**	0.061	0.968
3	Growth	Temp increased growth	**0.010**	0.302	0.845
	Production of new polyps	No effect	0.793	0.204	0.825
	Final weight/Final size	No effect	0.255	0.251	0.229
	F_v_/F_m_ on day 54	Nut increased F_v_/F_m_ while Temp decreased it	**<0.001**	**0.013**	**0.017**
	Survivorship curves	Nut increased survivorship while Temp decreased it	**<0.001**	0.076	**0.001**

### Elevated temperature and nutrient enrichment effects on larval survivorship (all preceding stages exposed to stressors; Experiment 1c)

After exposure of all early life processes (fertilization and embryo development) to the different treatments, survivorship of larvae in their first few days was significantly affected by temperature (p_Temperature_ < 0.001, Table 3). Larvae had highest survivorship (95 ± 4%) in treatments between 27–30°C, and at higher temperatures larval mortality increased significantly from 12 ± 18 at 30°C to 60 ± 18% at 32°C (Fig 3A). In contrast, nutrient enrichment and its interaction with temperature had no effect (p_Nutrients_ = 0.880, p_Temperature*Nutrients_ = 0.181, Table 3).

### Elevated temperature and nutrient enrichment effects on larval settlement (all preceding stages were exposed to stressors; Experiment 1d)

Settlement success of larvae that had developed from gametes under the different temperature and nutrient enrichment treatments was significantly affected only by temperature (p_Temperature_ < 0.001, Table 3). The highest settlement success was observed at 27°C (68 ± 13%), and settlement declined to 0% at 32°C (Fig 3B).

### Elevated temperature and nutrient enrichment effects on the settlement of 5-day-old larvae (no preceding stages exposed to treatment conditions; Experiment 2)

**Fig 4:**
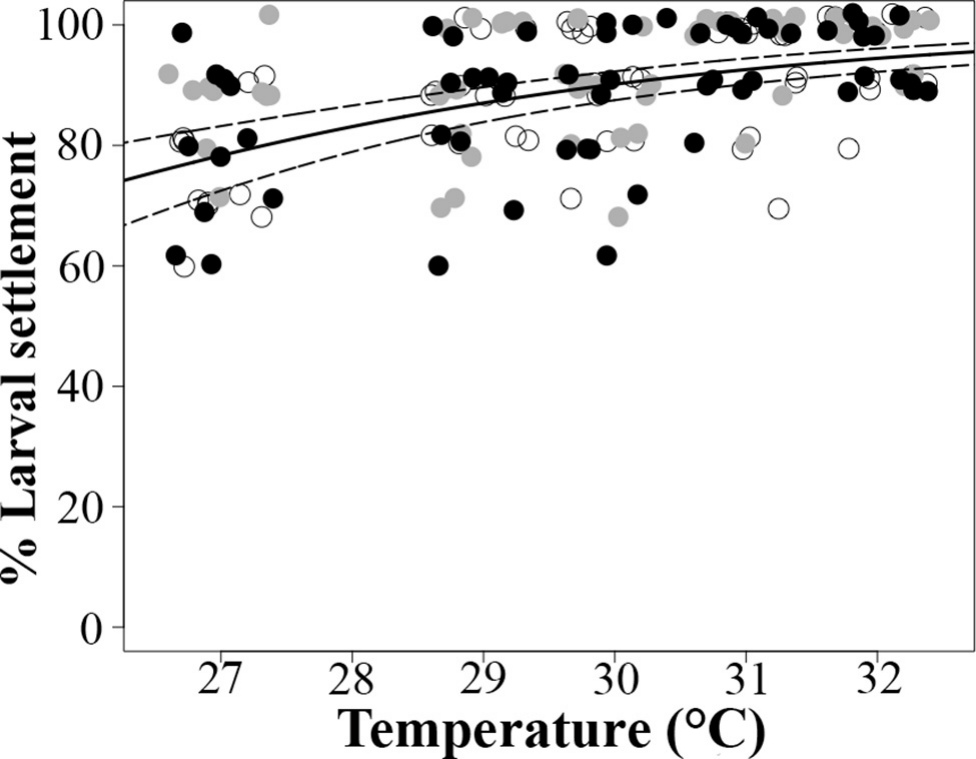
Proportion of 5 days old *Acropora tenuis* larvae, fertilized and reared under control conditions (27°C and FSW) but settled under different temperatures and nutrient enrichment [low (open circles), medium (grey circles), high (black circles); Experiment 2]. Control treatment: ‘low’ nutrient enrichment and at temperature = 27°C. Solid lines indicate fitted GLM trends, dashed lines are 95% confidence intervals. Individual points are jittered horizontally for clarity.

Settlement success of larvae fertilized and reared under control conditions (27°C and ambient nutrient levels) increased significantly with temperature (p_Temperature_ < 0.001, Table 3; Fig 4) being lowest (81 ± 11%) in the temperature control treatment (27°C) and highest (95 ± 5%) at 32°C, while no effect was found in response to nutrient enrichment or its interaction with temperature (p_Nutrients_ = 0.061, p_Temperature*Nutrients_ = 0.968, Table 3, Fig 4).

### Elevated temperature and nutrient enrichment effects on physiology and survivorship of 4-month-old coral juveniles (no preceding stages exposed to treatment conditions; Experiment 3)

**Fig 5:**
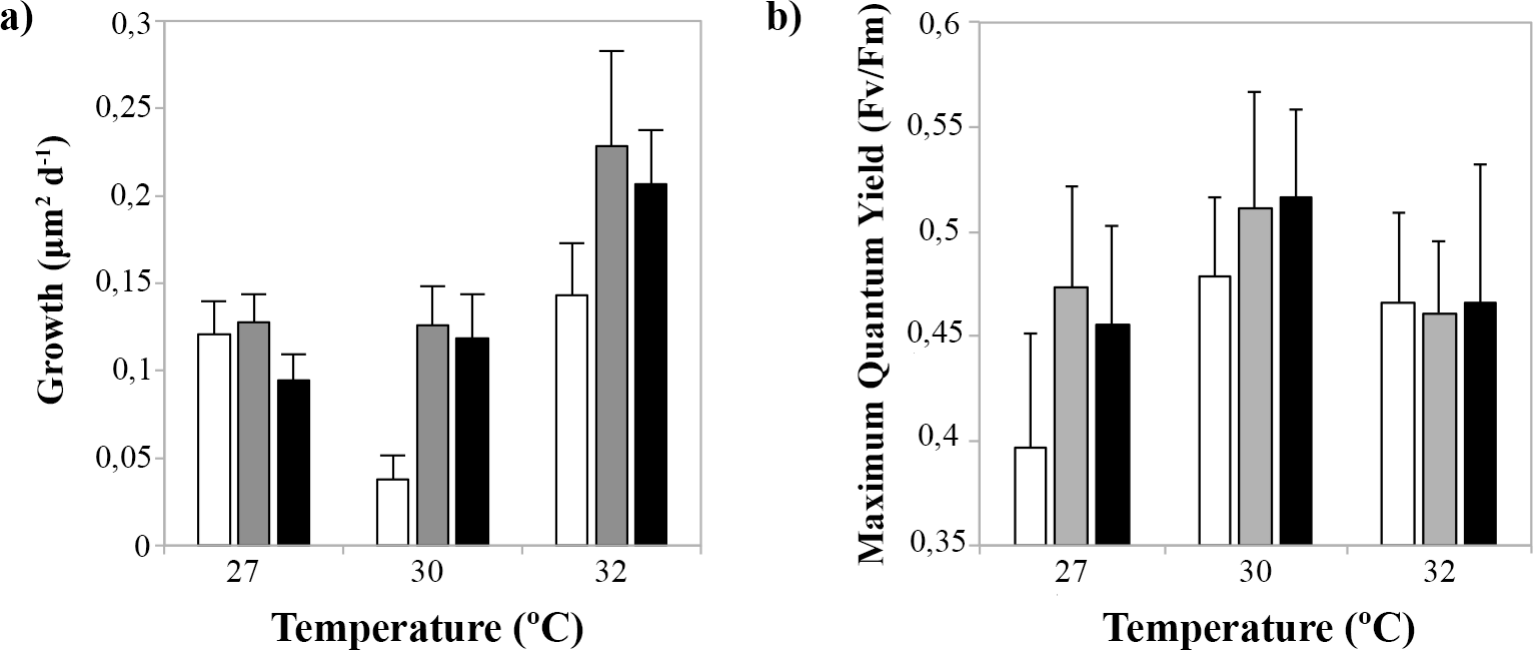
Four-month-old *Acropora tenuis* juveniles. **a) Growth rates (mean ± sd) under different temperatures and nutrient enrichment [low (white bars), medium (grey bars), high (black bars)], b) maximum quantum yields (F**_**v**_**/F**_**m**_**, mean ± sd) under different temperatures and nutrient enrichment (Experiment 3).** Control treatment: ‘low’ nutrient enrichment and at temperature = 27°C.

**Fig 6:**
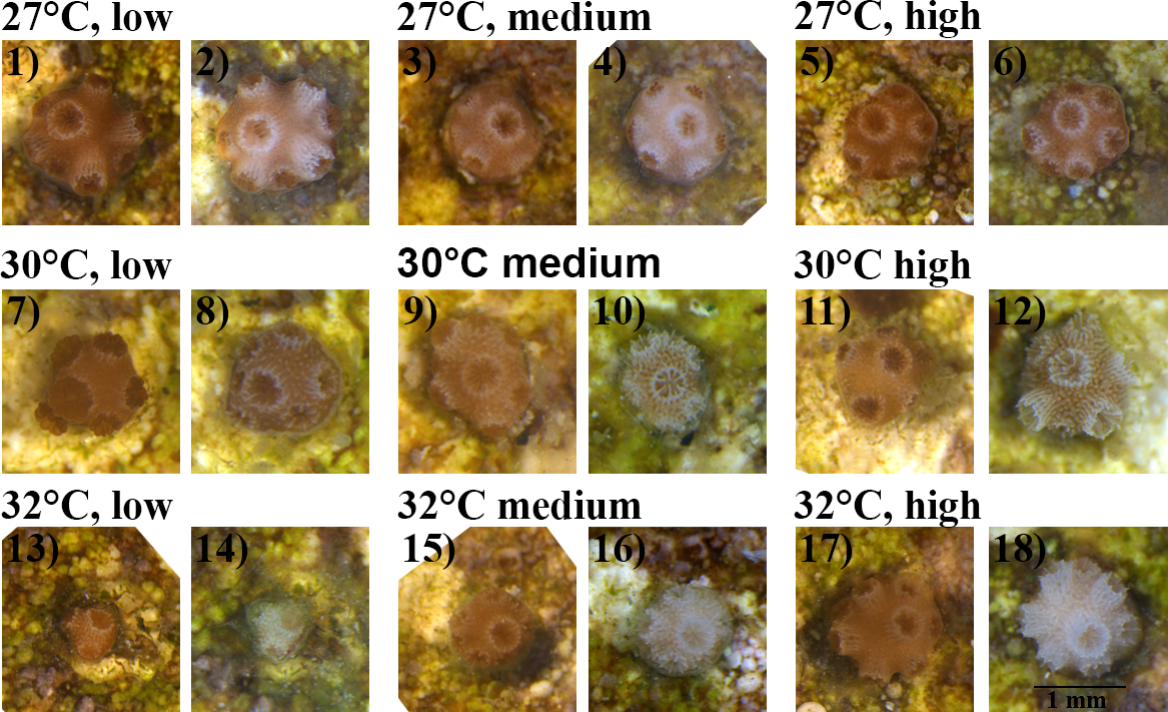
Images of juveniles on day 39 (odd numbers) and 59 (even numbers) of treatment exposure. Treatments consisted in three levels of nutrient enrichment (low, medium and high) and three temperatures (27, 30 and 32°C). Control treatment: ‘low’ nutrient enrichment and at temperature = 27°C.

**Fig 7:**
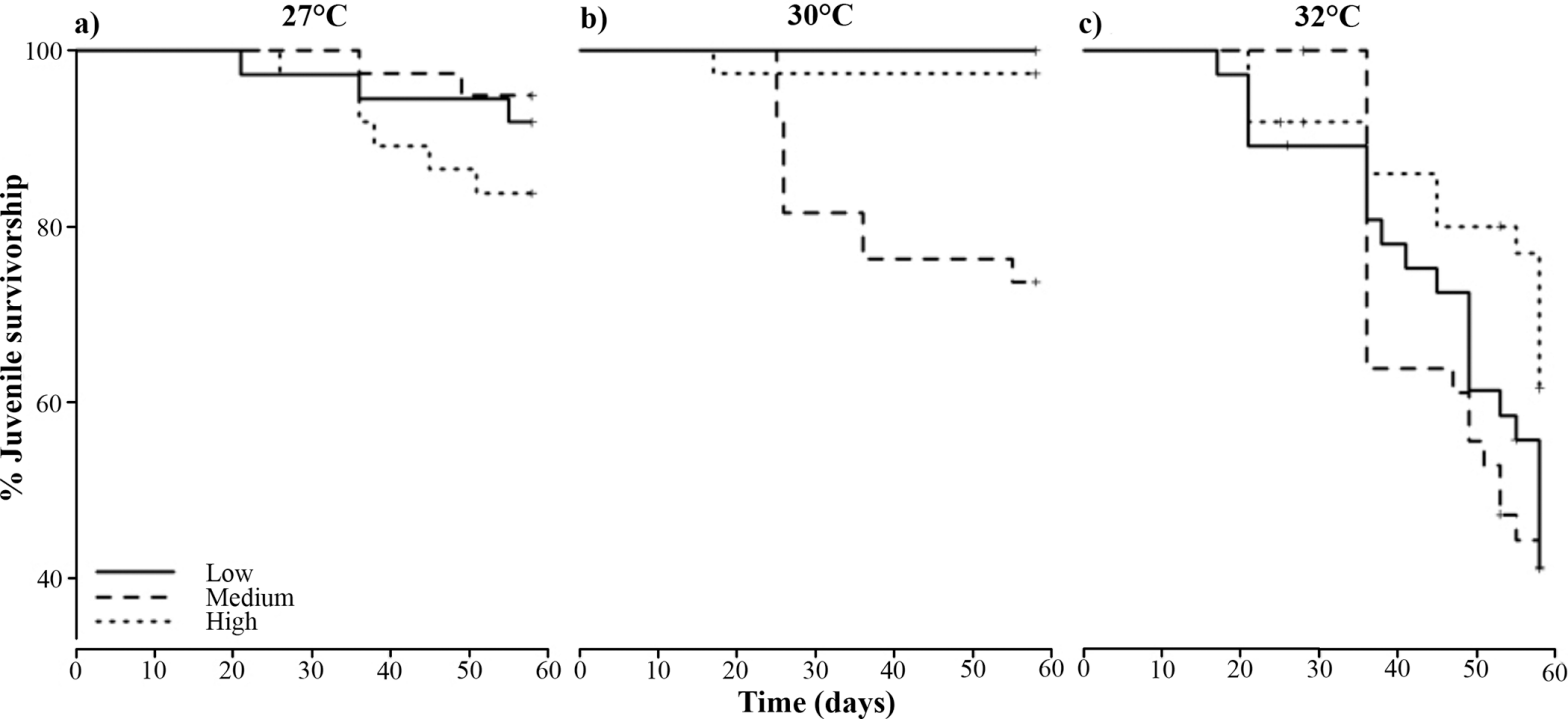
Survivorship curves of 4-month-old juveniles of *Acropora tenuis* that were exposed to nutrient enrichment [low (continuous line), medium (dashed line) and high (dotted line)] and temperature for 58 days. Control treatment: ‘low’ nutrient enrichment and at temperature = 27°C. Nutrient enrichment started on day one of the experiment, while heat stress started on day 21.

Growth rates (μm^2^ day^-1^) of coral juveniles changed significantly with temperature (p_Temperature_ = 0.010, Table 3, Fig 5A, Fig 6), but were unaffected by nutrient enrichment or a combination of the two factors (p_Nutrients_ = 0.302 and p_Temperature*Nutrients_ = 0.845, Table 3, Fig 5A). Juveniles exposed to 32°C exhibited the highest growth rates, having a 1.6-fold increase in area when compared to juveniles at 27°C (Fig 5A). However, there was no effect of temperature or nutrient enrichment on the rate of budding of new polyps (p_Temperature_ = 0.793 and p_Nutrients_ = 0.204, Table 3, S1 Fig), or on the relationship between their final skeletal dry weights and final size (p_Temperature_ = 0.255 and p_Nutrients_ = 0.251 respectively, Table 3). Photochemical efficiency (F_v_/F_m_) of the symbiotic algae was significantly affected by the combination of elevated temperature and nutrient enrichment in an interactive fashion (p_Temperature*Nutrients_ = 0.017, Table 3), and the combined effect of these factors was antagonistic (Fig 5B). Nutrient enrichment had a positive effect on F_v_/F_m_ until 30°C, but at 32°C (Fig 5B) the effect of the enrichment was counteracted by the negative effects of high temperatures, dropping F_v_/F_m_ values in the ‘medium’ and ‘high’ nutrient enrichment treatments to values similar to the ‘low’ nutrient enrichment treatment. Juvenile survival was also affected in an interactive fashion by temperature and nutrient enrichment (p_Temperature*Nutrients_ = 0.001, Table 3, Fig 7) and their interaction was multiplicatively antagonistic (GLM model). Survivorship at 27°C was slightly higher in the ‘low’ nutrient enrichment treatment (Fig 7A), while an increase to 30 and 32°C resulted in improved survivorship for juveniles exposed to ‘high’ nutrient enrichment (Fig 7B and 7C).

### Cumulative effects of elevated temperature and nutrient enrichment on total recruitment success

**Fig 8:**
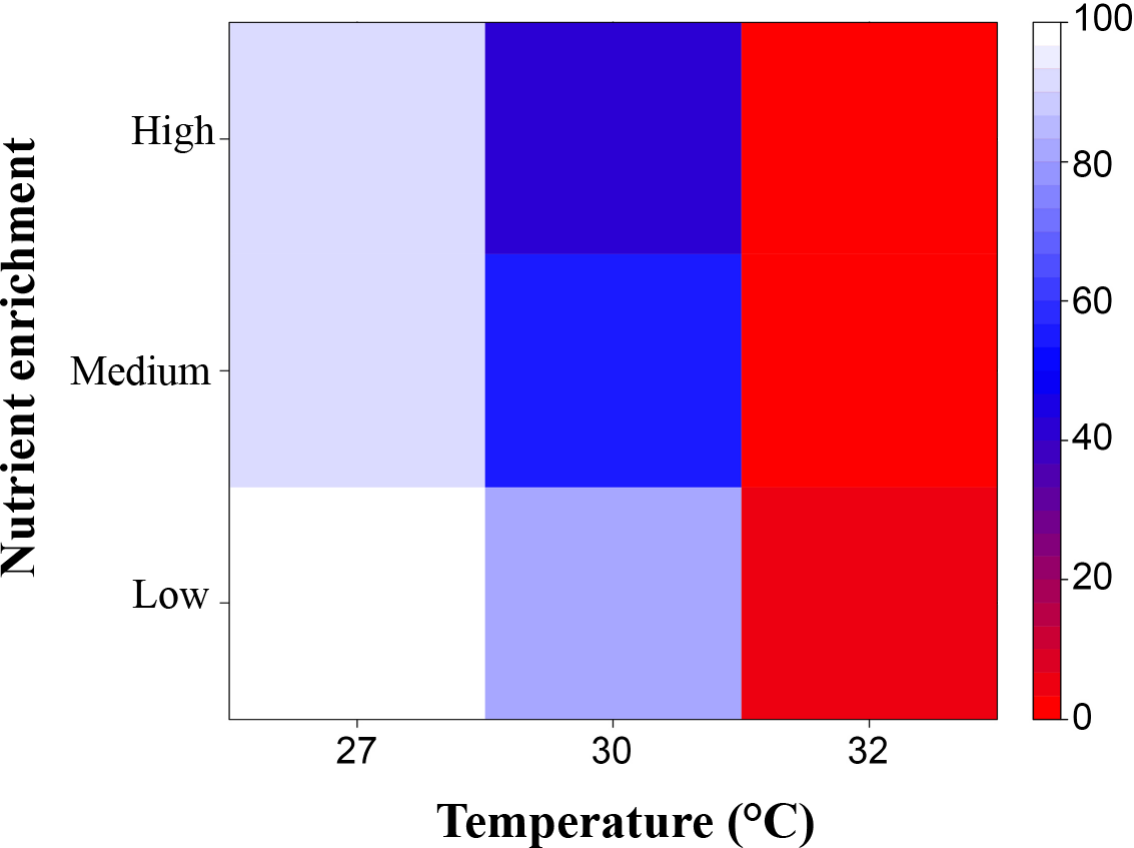
Total effect size of nutrient enrichment and temperature on recruitment success when the different stages (fertilization, embryo and larval development, settlement and 4-month-old juveniles) were equally exposed to contrasting temperatures and nutrient enrichment (low, medium and high). Control treatment: ‘low’ nutrient enrichment and at temperature = 27°C. Values between 0 and 100 indicate a negative effect of the treatment (*e*.*g*. 0 represents 0% survivorship), while a value of 100 indicates no effect of the treatment on the final stage considered (*i*.*e*. 100% survivorship).

When modelled together, the total effect of temperature increases and nutrient enrichment on recruitment success of *A*. *tenuis* was deleterious: it was reduced under exposure to the higher levels of either of the treatments, and was further reduced when the treatments were combined (Fig 8). Recruitment success declined compared with control values (normalised to 100%) to ≤ 50% at 30°C and ‘medium’ nutrient enrichment (Fig 8). Temperatures > 30°C lead to a < 50% reduction in recruitment success in all nutrient enrichment treatments.

## Discussion

This study illustrates that early life history stages of *A*. *tenuis* have different sensitivities to increased temperatures and nutrient enriched waters. Temperature and nutrient enrichment both reduced fertilization success, with their combined effects being additive for fertilization and synergistic for abnormal early embryo development respectively. Larval survivorship and settlement, and the growth rates of juveniles were only affected by increased temperatures, while survivorship of 4-month-old juveniles decreased in an antagonistic fashion when simultaneously impacted by increased temperature and nutrient enrichment. Modelling the effects of nutrient enrichment and heat stress together illustrated how changes in both local (water quality) and global factors (ocean warming) may interact to jointly decrease the success of successive early life history stages in corals. Our results also demonstrate serious consequences for overall recruitment if early life stages (from fertilization to recruit survival) are cumulatively exposed to elevated temperatures and/or nutrient enrichment.

This is the first study to test the effect of combined organic and inorganic nutrient enrichment on corals early life history stages; contrasting our work with previous studies which only tested the effects of elevated inorganic nutrients. Our experimental approach realistically preserves the stoichiometric composition of nutrients and trace elements present in seawater on river-exposed inshore reefs. This method has inherently higher natural variability among nutrient treatments than additions of specific forms of dissolved organic and inorganic nutrients, and also may affect other variables not measured here (microbial and plankton successions, enrichment or depletion of trace elements, accumulation of metabolites or pathogenic interactions). Consequently, our experimental design cannot tease apart effects of other variables potentially affected by the co-occurrence of nutrient enrichment and warming temperatures, limiting our capacity to fully elucidate mechanism affecting early life history stages. However, all these variables will also vary under natural conditions of eutrophication [70], and their impacts are likely to increase with increases of nutrient enrichment and temperature. These experiments therefore represent a valid first step to understand the interplay between nutrient enrichment and temperature and identify the most sensitive processes and stages to these stressors. Future research efforts should aim to understand the underlying mechanisms driving the observed impacts.

### Fertilization and embryo development under heat and nutrient enrichment stress

Reduced fertilization was observed in treatments with either high temperatures or nutrient enrichment (organic and inorganics), and effects were additive once treatments were combined (Experiment 1a). Previous single stress experiments have also found reduced fertilization under elevated temperatures [71, 72] or inorganic nutrients [31, 37]. Although our experimental design did not allow us to identify the individual mechanism(s) driving the impacts of combined stressors, results of previous single-factor studies suggest possible explanations. Elevated temperature is likely to impair coral fertilization through reduction sperm flagella motility, reducing the number of sperm-egg interactions [71]. In addition to possible molecular and biochemical impacts, nutrient enrichment is likely to foster the development of microbial communities that could be deleterious to gametes, a possibility that deserves further study. However, although other studies have also found elevated concentrations of dissolved inorganic nutrients reduce coral fertilization success [31, 33, 37]; the underlying mechanisms of nutrient toxicity on fertilization remain unknown. The additive effect of increased temperatures and nutrients indicates that enrichment is likely to exacerbate impacts on fertilization when high temperatures and coastal runoff coincide with coral spawning.

Abnormalities in early embryos were higher following exposure to high temperatures and nutrient enrichment, and these stressors acted synergistically when they co-occurred (Experiment 1b). Abnormal embryo development in corals has been described previously in response to temperature increases [39, 72–74] or in the presence of inorganic nutrients (however see [31, 33, 37]). Abnormalities could result from disruption of processes such as gene expression, cell rearrangement and differentiation, signalling pathways, arrested mitotic divisions, or impairment of functional enzymes or structural proteins [72, 75, 76]. The mechanism by which nutrients and elevated temperatures simultaneously affect embryo development at the ultrastructure level is unknown, although we hypothesize that it may be due to molecular, biochemical or microbial processes. Moreover, population-level implications of abnormal embryo development also remain unclear, as no studies so far have examined the ultimate fate of aberrant embryos. However, studies with other marine invertebrates [77–79] suggest that abnormalities result in energy depletion and higher mortality rates, which would clearly be deleterious for larval fitness and consequently for population maintenance.

### Larval survivorship and settlement under heat and nutrient enrichment stress

Embryos resulting from fertilization at high temperatures (>30°C) exhibited significantly reduced survivorship as they developed into planula larvae (Experiment 1c). The impacts of thermal stress on azooxanthellate larvae may be related to inhibition of their development or to sub-cellular damage, since decreases in larval cilia motility, pre-competency periods and survivorship have been observed after exposures to high temperatures [35, 38, 39, 80, 81]. In this study, the sensitivity of developing larvae to thermal stress may have been exacerbated by prior exposure during fertilization and early embryogenesis. The present study represents the first report of exposure of developing coral larvae to enriched seawater (Experiment 1c) and our results indicate that for *A*. *tenuis*, this life history stage may not be sensitive to nutrient enrichment.

Our results show that the effects of thermal stress on settlement success of *A*. *tenuis* depended on prior exposure during early development stages. Higher temperatures enhanced settlement success of larvae developed under control conditions (Experiment 2). Conversely, larvae developed from gametes and embryos that were also exposed to thermal stress exhibited reduced settlement and metamorphosis success (Experiment 1d). Larval settlement success has been observed to vary with intensity and frequency of temperature changes. Short-term (minutes to hours) exposure of larvae to higher temperatures have positive effects on settlement [81, 82], while longer exposures (days to months) can have negative effects ([34, 39], however see [56, 82]). Our results demonstrate that exposures to thermal stress and nutrient enrichment over fertilization and early (<2 h) embryogenesis can have significant flow-on impacts on larval fitness and function.

Positive effects of high temperatures on settlement success have been related to acceleration of metabolic rates in coral larvae [38, 82, 83]. However, increases in settlement success at elevated temperatures have also been accompanied by increases in post-settlement mortality [81, 82]. The consequences of accelerated settlement with increased temperature could be deleterious for population and metapopulation dynamics, since larval dispersal, connectivity and post-settlement survivorship have been observed to be compromised when larvae are exposed to thermal stress [84]. Consequently, thermal stress impacts on already competent larvae (Experiment 2) could have negative implications for coral reef resilience; however, they could also improve local settlement success.

### Single exposure of nutrients and temperature on 4-month-old juveniles

Physiological responses of 4-month-old juveniles of *A*. *tenuis* differed depending on the temperature and level of nutrient enrichment. For example, elevated temperatures had positive effects on growth, while the combination of both stressors affected photochemical performance (F_v_/F_m_) and survivorship of juveniles. While long-term impacts of thermal stress on adult corals are overwhelmingly negative [1], the effects of elevated organic and inorganic nutrients on adult corals are varied and can be negative, neutral or positive [85–88]. These results confirm that stressors have different mechanisms of action on metabolic processes and suggest trade-offs between processes that might determine the corals’ overall physiological performance under the co-occurrence of these stressors.

The maximum quantum yield of PSII in symbiotic zooxanthellae responded positively to nutrient enrichment at temperatures less than 32°C. This estimate is conservative since the most sensitive individuals died and were not included in the analysis. It has been proposed that enhancement of photosynthesis by dissolved and particulate organic nutrients occurs through the transfer of nitrogen from the host to the zooxanthellae and increases zooxanthellae division rates [89]. However, the positive effect of nutrient enrichment on photosystem efficiency was counteracted when temperatures reached 32°C, indicating that oxidative stress from high temperatures damaged PSII [90]. The occurrence of optimal F_v_/F_m_ values at 30°C is similar to findings previously reported for *A*. *tenuis* juveniles [86] and in adult corals of other species [87]. Improved performance of PSII at warmer (30°C) temperatures than at ambient (27°C) temperatures in *A*. *tenuis* associations may be related to symbiont clades having different reaction norms [88], presenting as greater photochemical performance and tolerance at high temperatures due to local adaptations [91]. However, values of F_v_/F_m_ at 32°C were similar to values obtained at 27°C despite marked differences in mortality in these two treatments, suggesting that F_v_/F_m_ was not a good predictor of the health status of coral juveniles in this experiment.

Among the physiological responses investigated for 4-month-old juveniles, growth and survivorship were affected to the greatest extent. Growth rates increased at the highest temperatures (32°C), in contrast to previous reports for other species [40, 92, 93]. Reduced growth rates in those studies were often linked to bleaching and the subsequent loss of energy derived from the zooxanthellae. While increasing temperatures had a clear negative effect on the survival of juveniles, the effects of nutrient enrichment varied, similar to a previous study with adult colonies of *A*. *millepora* [47].

### Cumulative effects of nutrient enrichment and temperature stress on early life history stages

There is mounting evidence that nutrients and heat stress produce adverse and long lasting effects on the reproductive output of corals. These stressors have induced reductions in the fecundity of colonies [94], fertilization and normal embryo developmental success [33, 72, 73], larval respiration rates [95], the duration of the larval pre-competency period [38] and larval survivorship [80]. The present study demonstrates that although some of the variables evaluated (i.e., larval settlement when exposed to the nutrient enrichment and temperature only during settlement and juvenile growth) had positive effects, the final outcome of the exposure of early life-history stages of *A*. *tenuis* to nutrient enrichment and temperature increases was a significant reduction in survivorship. The type of effect (additive vs synergistic or antagonistic) of nutrient enrichment and high temperatures varied in direction and intensity between the different early life history stages of *A*. *tenuis*. Nonetheless, we found temperature increase to be the main driver of detrimental impacts on recruitment success, with nutrient enrichment subtly increasing the impacts at the highest temperatures.

Our results indicate that the early life history stages of corals can be sensitive to temperature and that this effect is more pronounced (i) in early development (gametes and early embryos) and (ii) in the presence of nutrient-enriched water. This study shows that nutrient enrichment increases the impact of thermal stress on *A*. *tenuis* by compromising their replenishment capacity through reductions in survivorship of sexually produced individuals. Even without heat stress, exposure to nutrient enrichment will have a strong detrimental effect on the earliest development processes of hard corals (fertilization and embryo development), compromising later larval settlement and juvenile survivorship. Future research studies should be focused on understanding the possible mechanism of action of the individual and simultaneous occurrence of nutrient enrichment and temperature stress on corals early life history stages, including detailed analysis of structural and metabolic pathways changes during exposures. Management strategies focused on water quality improvements by reducing the input of fertilizers will not only prevent coral mortality and macroalgae blooms [96], but they will also enhance reef resilience by improving the thermal tolerance of early life history stages of some inshore coral species.

## Supporting Information

S1 Fig Four-month-old *Acropora tenuis* juveniles production of new polyps (mean ± sd) between day 4 and 59 of the experiment under different temperatures and nutrient enrichment [low (white bars), medium (grey bars), high (black bars)]. Control treatment: ‘low’ nutrient enrichment and at temperature = 27°C.
(TIF)

S1 Table **Temperatures (°C) during the incubation of the modified FSW with nutrient enrichment (Nut) and during Experiments 1 (a, b, c and d), 2 and 3 (incubation period in Experiment 3 corresponds to the exposure to nutrient enrichment during 20 days before starting the temperature stress).** Values shown are means ± sd.
(DOCX)

S2 Table **GLM results showing the effects of temperature (Temp) and nutrient enrichment (Nut) on i) fertilization success (Experiment 1a), embryo development (Experiment 1b), larval development (Experiment 1c) and larval settlement (Experiment 1d), and ii) juvenile growth, production of new polyps, final weigh/final size, F_v_/F_m_, survivorship curves (Experiment 3) of *Acropora tenuis*.** Temperature and nutrient enrichment were considered as fixed factors. Significance at p<0.05 is shown in bold. Df: degrees of freedom.
(DOCX)
