# Supplementary figures and images for: Cumulative Effects of Nutrient Enrichment and Elevated Temperature Compromise the Early Life History Stages of the Coral Acropora tenuis
Source: PLoS One. 2016 Aug 30;11(8):e0161616. doi: 10.1371/journal.pone.0161616 (PMC5004850; doi:10.1371/journal.pone.0161616)

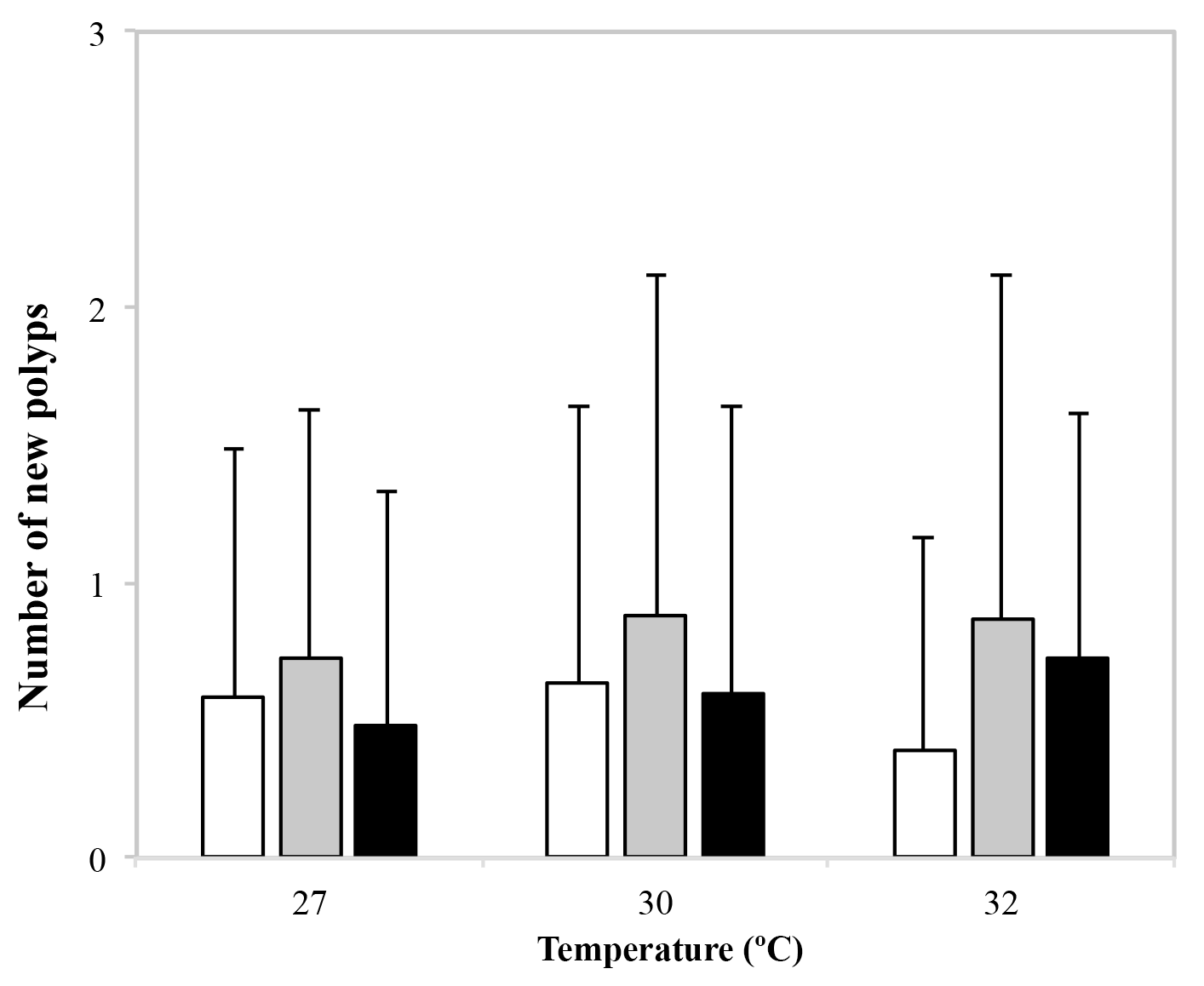

Supplement: S1 Fig — Control treatment: ‘low’ nutrient enrichment and at temperature = 27°C. (TIF) [file pone.0161616.s001.tif]
