# Supplementary material for: Cumulative Effects of Nutrient Enrichment and Elevated Temperature Compromise the Early Life History Stages of the Coral Acropora tenuis
Source: PLoS One. 2016 Aug 30;11(8):e0161616. doi: 10.1371/journal.pone.0161616 (PMC5004850; doi:10.1371/journal.pone.0161616)
Supplement: S1 Table — Temperatures (°C) during the incubation of the modified FSW with nutrient enrichment (Nut) and during Experiments 1 (a, b, c and d), 2 and 3 (incubation period in Experiment 3 corresponds to the exposure to nutrient enrichment during 20 days before starting the temperature stress). Values shown are means ± sd. (DOCX) [file pone.0161616.s002.docx]

**S1 Table:** Temperatures (ºC) reached during the incubation of the modified FSW with organic nutrient enrichment (Nut) and during Experiments 1 (a, b, c and d), 2 and 3 (incubation period in Experiment 3 corresponds to the exposure to Nut during 20 days before starting the temperature stress). Values shown are means ± standard deviations.

|  | **Expected Temperature** | **Fertilization (Experiment 1a and b)** | **Larvae development (Experiment 1c)** | **Settlement (Experiment 1d and 2)** | **Juveniles (Experiment 3)** |
| --- | --- | --- | --- | --- | --- |
| **Incubation of Nut** | 27ºC | 27.23 ± 0.36ºC | 27.40 ± 0.17ºC | 27.45 ± 0.15ºC | 27.10 ± 0.10ºC |
|  | 29ºC | 28.64 ± 0.65ºC | 28.49 ± 0.70ºC | 29.76 ± 0.081ºC |  |
|  | 30ºC | 30.32 ± 0.70ºC | 30.62 ± 0.61ºC | 30.28 ± 0.18ºC | 27.13 ± 0.25ºC |
|  | 31ºC | 31.08 ± 0.18ºC | 30.88 ± 0.70ºC | 31.60 ± 0.36ºC |  |
|  | 32ºC | 32.32 ± 0.44ºC | 32.52 ± 0.35ºC | 32.77 ± 0.10ºC | 27.15 ± 0.43ºC |
| **Experiment** | 27ºC | 27.55 ± 0.13ºC | 27.48 ± 0.15ºC | 27.39 ± 0.15ºC | 27.07 ± 0.18ºC |
|  | 29ºC | 29.35 ± 0.19ºC | 29.44 ± 0.31ºC | 29.05 ± 0.62ºC |  |
|  | 30ºC | 30.58 ± 0.10ºC | 30.54 ± 0.48ºC | 30.49 ± 0.06ºC | 29.98 ± 0.16ºC |
|  | 31ºC | 30.99 ± 0.18ºC | 31.44 ± 0.49ºC | 31.25 ± 0.68ºC |  |
|  | 32ºC | 32.76 ± 0.05ºC | 32.76 ± 0.11ºC | 32.70 ± 0.05ºC | 31.94 ± 0.20ºC |
