# Supplementary material for: Cumulative Effects of Nutrient Enrichment and Elevated Temperature Compromise the Early Life History Stages of the Coral Acropora tenuis
Source: PLoS One. 2016 Aug 30;11(8):e0161616. doi: 10.1371/journal.pone.0161616 (PMC5004850; doi:10.1371/journal.pone.0161616)
Supplement: S2 Table — GLM results showing the effects of temperature (Temp) and nutrient enrichment (Nut) on i) fertilization success (Experiment 1a), embryo development (Experiment 1b), larval development (Experiment 1c) and larval settlement (Experiment 1d), and ii) juvenile growth, production of new polyps, final weigh/final size, Fv/Fm, survivorship curves (Experiment 3) of Acropora tenuis. Temperature and nutrient enrichment were considered as fixed factors. Significance at p<0.05 is shown in bold. Df: degrees of freedom. (DOCX) [file pone.0161616.s003.docx]

**S2 Table:** Effects of temperature (Temp) and organic nutrient enrichment (Nut) on i) fertilization success (Experiment 1a), embryo development (Experiment 1b), larval development (Experiment 1c) and larval settlement (Experiment 1d), and ii) juvenile growth, production of new polyps, final weigh/final size, F_v_/F_m_, survivorship curves (Experiment 3) of *Acropora tenuis*. Temperatuture (Temp) and organic nutrient enrichment (Nut) were considered as fixed factors. Significance at p<0.05 is shown in **bold**. Df: degrees of freedom.

i)

| **Experiment** | **Factor** | **Df** | **Deviance** | **F-value** | **p-value** |
| --- | --- | --- | --- | --- | --- |
| Fertilization (1a) | Temp | 1 | 1600.20 | 17.462 | **<0.001** |
|  | Nut | 2 | 1616.20 | 9.245 | **<0.001** |
|  | Temp:Nut | 2 | 1330.10 | 0.954 | 0.389 |
| Embryo development (1b) | Temp | 1 | 1207.73 | 173.532 | **<0.001** |
|  | Nut | 2 | 494.46 | 10.128 | **<0.001** |
|  | Temp:Nut | 2 | 512.75 | 12.975 | **<0.001** |
| Larval survivorship (1c) | Temp | 1 | 892.26 | 118.899 | **<0.001** |
|  | Nut | 2 | 375.61 | 0.127 | 0.880 |
|  | Temp:Nut | 2 | 374.50 | 1.739 | 0.181 |
| Larval settlement (1d) | Temp | 1 | 808.76 | 65.584 | **<0.001** |
|  | Nut | 2 | 459.38 | 0.050 | 0.951 |
|  | Temp:Nut | 2 | 458.84 | 0.111 | 0.895 |
| Larval settlement (2) | Temp | 1 | 241.53 | 43.052 | **<0.001** |
|  | Nut | 2 | 200.11 | 2.828 | 0.061 |
|  | Temp:Nut | 2 | 193.84 | 0.032 | 0.968 |

ii)

| **Experiment** | **Factor** | **Df** | **Likelihood ratio test** | **p-value** |
| --- | --- | --- | --- | --- |
| Growth | Temp | 2 | 9.175 | **0.010** |
|  | Nut | 2 | 2.391 | 0.302 |
|  | Temp:Nut | 4 | 3.447 | 0.485 |
| Production of new polyps | Temp | 2 | 0.463 | 0.793 |
|  | Nut | 2 | 3.175 | 0.204 |
|  | Temp:Nut | 4 | 1.504 | 0.825 |
| Final weight/Final size | Temp | 2 | 0.726 | 0.696 |
|  | Nut | 2 | 2.259 | 0.323 |
|  | Temp:Nut | 4 | 5.371 | 0.251 |
| F_v_/F_m_ | Temp | 2 | 16.334 | **<0.001** |
|  | Nut | 2 | 8.617 | **0.013** |
|  | Temp:Nut | 4 | 11.931 | **0.017** |
